# Clinical Features and Outcomes of Invasive Breast Cancer: Age-Specific Analysis of a Modern Hospital-Based Registry

**DOI:** 10.1200/JGO.19.00034

**Published:** 2019-07-01

**Authors:** Ji-Yeon Kim, Danbee Kang, Seok Jin Nam, Seok Won Kim, Jeong Eon Lee, Jong Han Yu, Se Kyung Lee, Young-Hyuck Im, Jin Seok Ahn, Eliseo Guallar, Juhee Cho, Yeon Hee Park

**Affiliations:** ^1^ Sungkyunkwan University School of Medicine, Seoul, Republic of Korea; ^2^ Johns Hopkins Bloomberg School of Public Health, Baltimore, MD

## Abstract

**PURPOSE:**

We evaluated the clinical features and outcomes of invasive breast cancer (BC) among different age groups by analyzing a modern BC registry including subtypes and treatment information.

**METHODS:**

This was a retrospective cohort study of 6,405 women aged 18 years or older with pathologically confirmed stage I, II, or III BC who underwent curative surgery followed by adjuvant therapy at a university-based hospital in Seoul, South Korea, between January 2003 and December 2011. The study end point was all-cause mortality. We used Cox proportional hazards models and hazard ratios (HRs) with 95% CIs calculated after adjusting for age, body mass index, stage, subtype, and treatment, including type of surgery and use of chemotherapy, radiation therapy, hormone therapy, and targeted therapy.

**RESULTS:**

During 36,360 person-years of follow-up (median follow-up: 5.45 years; interquartile range, 4.3-7.1), 256 deaths were reported (mortality rate, 7.0/1,000 person-years). The adjusted HR for all-cause mortality was higher in patients older than 40 years (HR, 2.03; 95% CI, 1.44 to 2.87) and older than 60 years (HR, 2.35; 95% CI, 1.63 to 3.39) than in patients aged 40 to 49 years. Across age groups, advanced stage at diagnosis, luminal type as well as triple-negative BC, and not receiving adjuvant treatment were associated with increased risk of mortality.

**CONCLUSION:**

A strong J-shaped relationship was observed between age and mortality, indicating worse clinical outcomes in young and old patients. This study suggested a possible benefit of personalized BC screening examination and precise and active treatment strategies to reduce BC-related mortality.

## INTRODUCTION

Breast cancer (BC) is the most common type of cancer in women worldwide, with approximately 1,670,000 new cases globally.^1^ Although 90% of patients with BC survive for over 5 years, up to 10% of patients experience disease recurrence and die of disease progression after curative surgery.^2,3^ TNM stage, tumor grade, estrogen receptor (ER), progesterone receptor (PR), and human epidermal growth factor receptor 2 (HER2) status are major predictive markers for recurrence^4^; they have been incorporated into management guidelines and are used to personalize treatment regimens with targeted agents. Moreover, age at diagnosis,^5^ alcohol consumption,^6^ smoking,^7^ and obesity^8^ have been associated with prognosis in some studies, but young age at diagnosis was consistently associated with poor prognosis.^9,10^ Most studies of clinical outcomes and prognostic factors of BC have been conducted in Western countries before the introduction of modern tumor subtyping and targeted treatments. Moreover, the results from Western studies may not be applicable to Asian women, because of the major differences in clinical characteristics of BC in these women.

BC is the second most common cancer in Korea after thyroid cancer (crude incidence rate, 72.1 per 100,000 people in 2014).^11^ Up to 50% of Korean patients with BC receive the diagnosis before 50 years of age, and most Korean women with BC are premenopausal, one of the risk factors for disease progression and poor clinical outcomes.^9^ Some studies have investigated the clinical characteristics and outcomes of Korean patients with BC by age; however, these studies only evaluated a specific age group—either the very young (< 40 years) or very old (> 70 years).^12,13^ In addition, like previous studies in Western countries, they had limited information about subtype and detailed treatments, including hormone or targeted therapy.^13-16^ In this study, we aimed to evaluate the clinical features and outcomes of invasive BC among different age groups by analyzing a large hospital-based BC registry including information on modern subtyping and detailed treatment. We specifically hypothesized that the clinical features and outcomes would be different depending on the patient’s age at diagnosis.

## METHODS

### Study Population

This was a retrospective cohort study of women at least 18 years old with pathologically confirmed stage I, II, or III BC (N = 6,692) who underwent curative surgery followed by adjuvant systemic therapy at Samsung Medical Center, Seoul, Korea, between January 2003 and December 2011. We excluded patients who had missing information on menopausal status (n = 115) or subtype (ER, n = 10; PR, n = 9; and HER2, n = 181). Because study participants could have more than one exclusion criterion, the final sample size was 6,405. The Institutional Review Board of the Samsung Medical Center approved this study and waived the requirement for informed consent because we used only deidentified data routinely collected during clinical care.

### Measurements

Detailed information on surgery, adjuvant chemotherapy, radiotherapy, hormone therapy, and targeted therapy were obtained from electronic medical records. Body mass index (BMI) was calculated as weight in kilograms divided by height in meters squared at the time of BC diagnosis. Women were considered postmenopausal if they were amenorrheic for at least 12 months, had a prior bilateral oophorectomy, or were age 60 years or older.^4^

Pathologic stage was based on the criteria of the American Joint Committee on Cancer.^17^ Two pathologists with 13 and 17 years of experience, respectively, reviewed and determined the primary tumor characteristics on the basis of size, axillary nodal status, resection margin, and receptor status (ER, PR, and HER2) by immunohistochemical staining. ER positivity and PR positivity were defined as an Allred score of 3 to 8, on the basis of immunohistochemical staining with antibodies against ER (Immunotech, France) and PR (Novocastra, UK), respectively. HER2 status was evaluated using the appropriate antibody (Dako, Carpinteria, CA) and/or silver in situ hybridization. HER2 grades 0 and 1 indicated a negative result, and grade 3 indicated a positive result. Amplification of HER2 was confirmed by silver in situ hybridization for results of 2+. Triple negative BC (TNBC) was defined as BC with negative ER expression, PR expression, and HER2 overexpression.

The study end point was all-cause mortality. The secondary end point was distant recurrence, including soft tissue or nodal metastases in distant sites, bone metastases, visceral metastases in other organs, and diffuse intra-abdominal metastases.

### Statistical Analysis

For all-cause mortality, patients were included in the study at the time of diagnosis and were followed up until death or the end of the study period (May 31, 2016). To determine the clinical features associated with mortality, we used Cox proportional hazards models and hazard ratios (HRs) with 95% CIs calculated after adjusting for age, BMI, stage, subtype, and treatment, including type of surgery and use of chemotherapy, radiation therapy, hormone therapy, and targeted therapy. With regard to distant recurrence, patients were followed until the first recorded evidence of treatment failure or until the last follow-up visit for those alive without recurrence. To account for competing risks due to mortality, we fitted a proportional subdistribution hazards regression model using the Fine and Gray regression model^18^ with death as the competing event. All reported *P* values were two sided, and the significance level was set at 0.05. Statistical analyses were performed using STATA, version 14 (StataCorp LP, College Station, TX).

## RESULTS

The median age at BC diagnosis was 48.6 (interquartile range, 42-54) years; approximately 16.7%, 42.5%, 26.5%, and 14.3% of women were younger than 40 years old, 40 to 49 years old, 50 to 59 years old, and older than 60 years, respectively; and 37.2% of patients were postmenopausal (Table 1). Altogether, 12.7% of patients were diagnosed with stage III BC, and those younger than 40 years were more likely to be diagnosed with stage III (14.6%) than were older patients (*P* < .001). The proportion of BC subtype was significantly different by age groups: ER+ or PR+ BC and HER2− BC commonly occurred in women in the 40 to 49 years age group (70.1%), whereas ER−, PR−, and HER2− BC (ie, TNBC) commonly occurred in women younger than 40 years (21.6%) (Table 1). In terms of treatment, 72.3%, 75.5%, 98.8%, and 46.7% of patients received chemotherapy, radiation therapy, hormone therapy, and targeted therapy, respectively; and patients older than 60 years were less likely to receive chemotherapy or radiotherapy (Table 1).

**TABLE 1:**
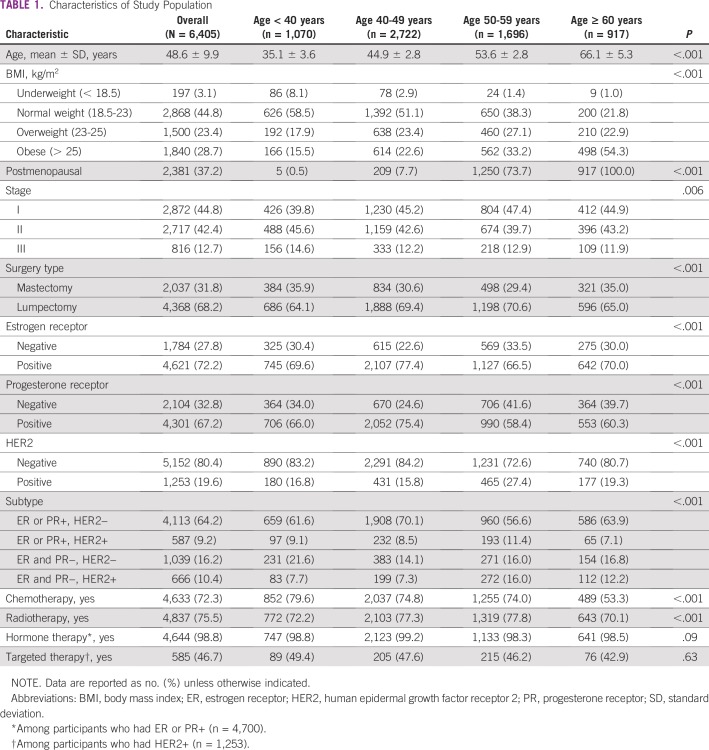
Characteristics of Study Population

**FIG 1:**
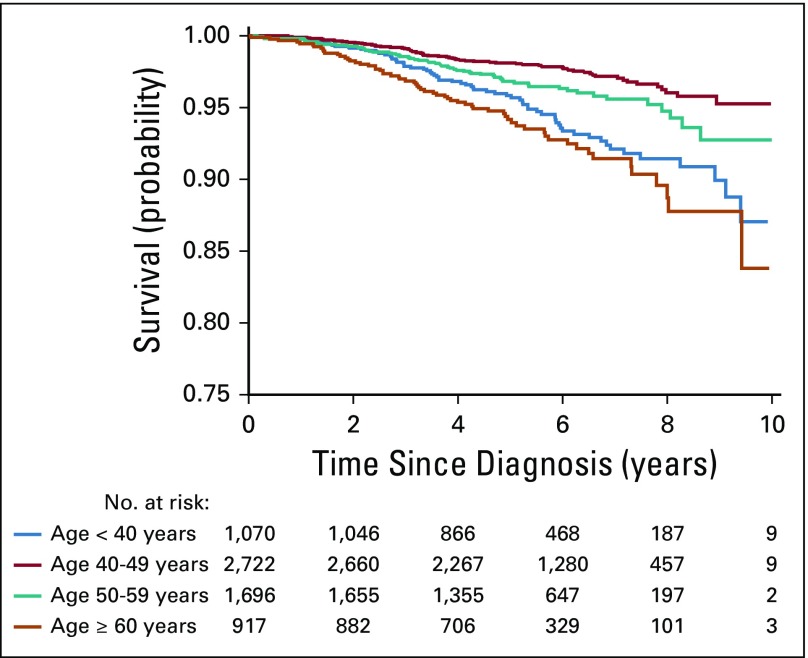
Cumulative probability of surviving according to age.

**FIG 2:**
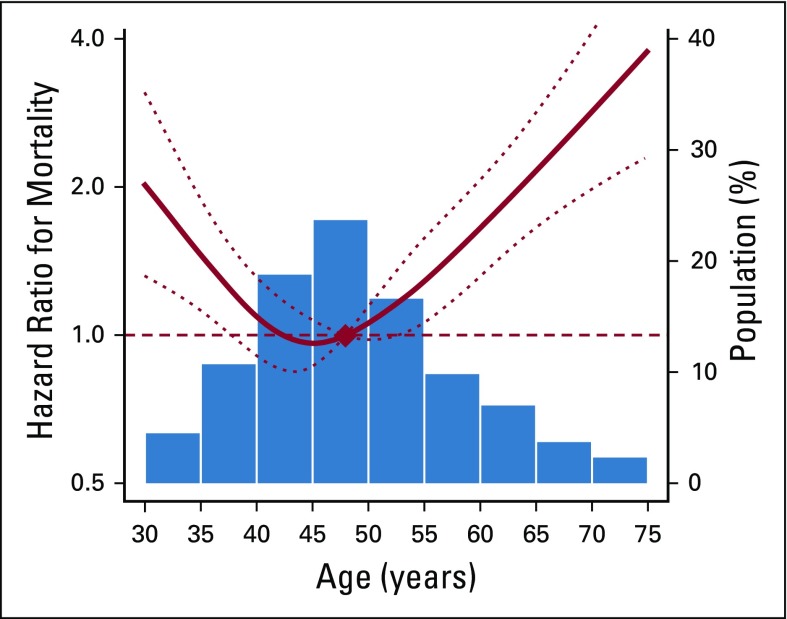
Hazard ratios for overall mortality by age. Curves represent adjusted hazard ratios (solid lines) and their 95% CIs (dotted lines) on the basis of restricted cubic splines for age with knots at the fifth, 35th, 65th, and 95th percentiles (34, 44, 51, and, 67 years, respectively) of their sample distributions. The reference values (diamond dot) were set at the 50th percentile (47 years). Adjusted for age, body mass index, stage, and treatment, including surgery, chemotherapy, and radiotherapy.

After 36,360 person-years of follow-up (median follow-up: 5.45 years; interquartile range, 4.3-7.1), 256 deaths were reported (mortality rate, 7.0 per 1,000 person-years). In the entire follow-up, the cumulative probability of overall survival was consistently lower in participants younger than 40 years and older than 60 years compared with those 40 to 49 years old (Fig 1). In spline regression models, there was a strong J-shaped association between age and mortality (Fig 2). After adjusting for BMI, stage, and treatment including surgery, chemotherapy, and radiotherapy, the HR for all-cause mortality was significantly higher in patients younger than 40 years (HR, 2.03; 95% CI, 1.44 to 2.87) and in patients older than 60 years (HR, 2.35; 95% CI, 1.63 to 3.39) than in those 40 to 49 years old (Table 2).

**TABLE 2:**
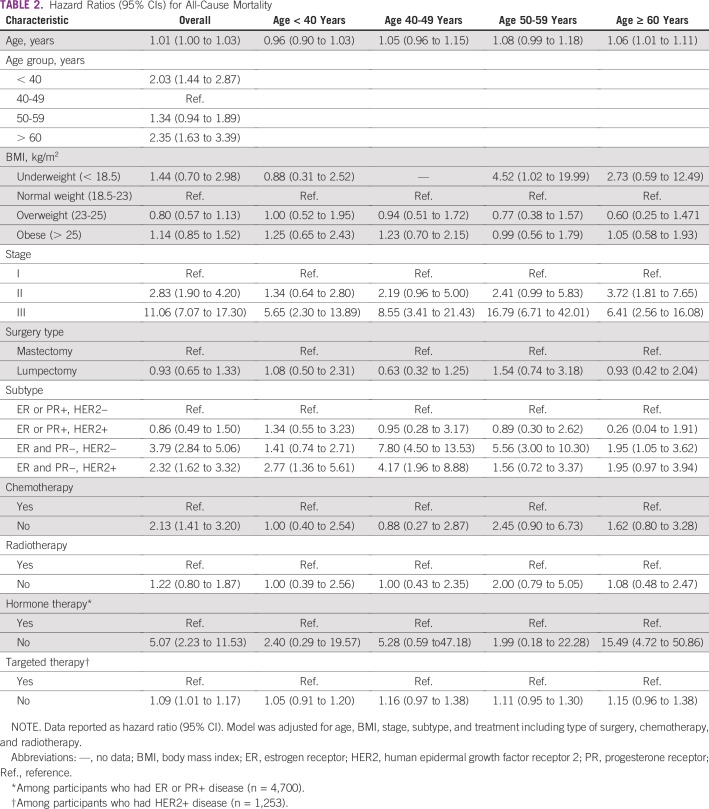
Hazard Ratios (95% CIs) for All-Cause Mortality

Obesity was not associated with increased mortality, but underweight was significantly associated with mortality in patients 50 to 59 years old (HR, 4.52; 95% CI, 1.02 to 19.99). Although underweight also increased risk of mortality in patients older than 60 years (HR, 2.73; 95% CI, 0.59 to 12.49), it was not a statistically significant factor.

Multivariable-adjusted HRs were significantly higher in patients with stage II (HR, 2.83; 95% CI, 1.90 to 4.20) and stage III (HR, 11.06; 95% CI, 7.07 to 17.30) BC than in those with stage I. With regard to subtype, multivariable-adjusted HRs were higher in patients with TBNC (HR, 3.79; 95% CI, 2.84 to 5.06) and ER−, PR−, and HER2+ BC (HR, 2.32; 95% CI, 1.62 to 3.32) than in those with ER+ or PR+ and HER2− BC. The positive association among stage, TNBC, and mortality was consistent in all age groups. Patients who did not receive chemotherapy (HR, 2.13; 95% CI, 1.41 to 3.20), hormone therapy (HR, 5.07; 95% CI, 2.23 to 11.53), and targeted therapy (HR, 1.09; 95% CI, 1.01 to 1.17) were more likely to die than those who received each specific therapy. Especially, among patients older than 60 years, those who did not receive hormone therapy had a 15-times higher risk of dying compared with patients receiving hormone therapy (HR, 15.49; 95% CI, 4.72 to 50.86; Table 2).

**FIG 3:**
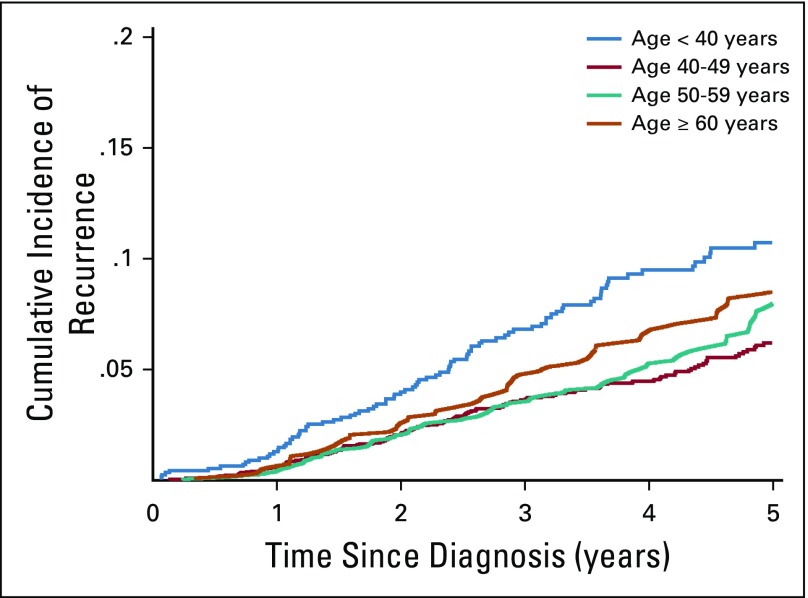
Cumulative incidence function of recurrence according to age.

The cumulative distant recurrence rates were consistently higher in participants younger than 40 years (HR, 1.68; 95% CI, 1.29 to 2.18) compared with those 40 to 49 years old (Table 3; Fig 3). In multivariable models with mortality as a competing risk, advanced stage was associated with recurrence in all age groups. Patients with TNBC (HR, 2.24; 95% CI, 1.75 to 2.87) and ER−, PR−, and HER2+ BC (HR, 1.50; 95% CI, 1.09 to 2.06) had higher risk of distant recurrence than those with ER+ or PR+ BC and HER2− BC. Moreover, patients who did not receive hormone therapy had a higher risk of distant recurrence (HR, 2.61; 95% CI, 1.13 to 6.00) than those who received hormone therapy.

**TABLE 3:**
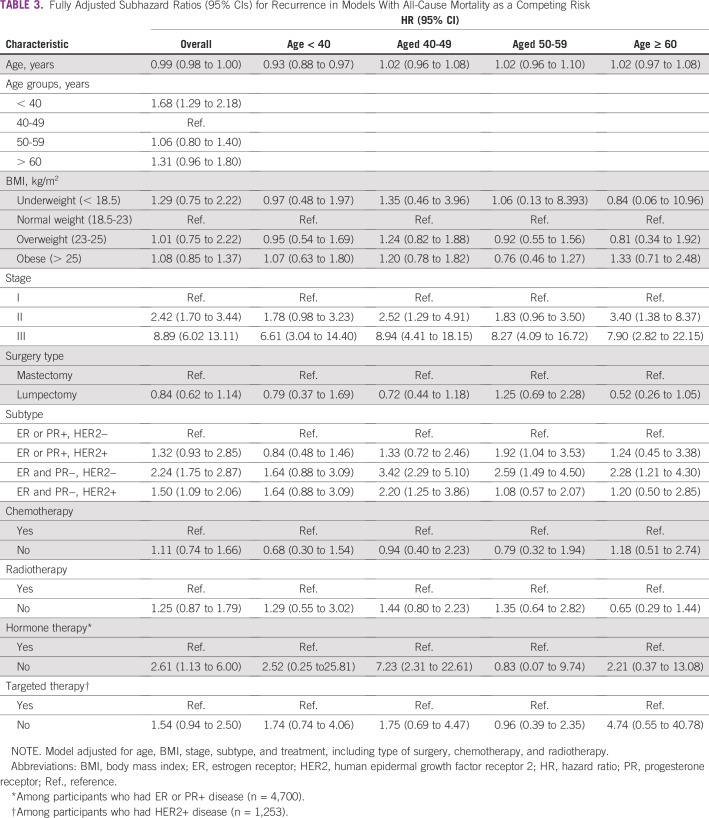
Fully Adjusted Subhazard Ratios (95% CIs) for Recurrence in Models With All-Cause Mortality as a Competing Risk

## DISCUSSION

By analyzing this modern BC registry, we found a strong J-shaped association between age and mortality. The HR for all-cause mortality was significantly higher in patients younger than 40 years and older than 60 years than those 40 to 49 years old, after adjusting for all other risk factors. Besides age group, advanced stage at diagnosis, luminal type including TNBC, and not receiving chemotherapy, hormone therapy, and targeted therapy were associated with all-cause mortality. Different clinical features were associated with increased risk of all-cause mortality and distant recurrence, depending on age groups.

In all age groups, advanced cancer stage was significantly associated with all-cause mortality and distant recurrence, emphasizing the importance of BC screening and early detection. There was clear evidence that regular screening can help detect early-stage cancer^19^ and screening examination provides a significant decrease (20% to 35%) in BC mortality.^20^ In our study, patients younger than 40 years were more likely to be diagnosed with stage III BC than were older patients, resulting in relatively higher mortality. In Korea, like most other countries, BC screening is provided to women older than 40 years.^21,22^ Therefore, women younger than 40 years would have less chance of undergoing BC screening such as mammography or breast ultrasonography than would older patients who were recommended for routine screening. In fact, many women at an average risk of BC are offered relatively little opportunity to discuss and initiate mammographic screening before the age of 50 years.^23^ Although regular screening or clinical examination of women younger than 40 years remains controversial, personalized approaches to BC screening have been discussed. Now a clinical trial in the United States is examining efficacy of screening patients at an earlier age, regular performance of mammograms, and continuation of screening until women are older, on the basis of a model including personal history, family history, and genetic testing.^24^ It would be necessary to conduct similar trials with Asian women, considering that their clinical features are different from those of Western women. In our study, the proportion of patients with stage I BC who were older than 60 years was relatively small, which might be associated with the higher mortality observed in individuals that age group. Previous studies found that patients 60 to 69 years old were 0.61 times less likely to undergo screening mammography than those 40 to 49 years old.^25^ Although we did not have screening information, the lower proportion of patients with stage I BC who were older than 60 years might be due to the lower screening rate. Older women should be encouraged to undergo regular screening for the early detection of cancer, which would facilitate early treatment and improvement of prognosis.

Beside advanced stage at diagnosis, young patients with BC had worse clinical outcomes, partly because of the over-representation of more aggressive subtypes such as TNBC or HER2+ BC.^26^ In fact, in our study, higher proportions of women with stage II and III BC and TNBC were younger than 40 years. Considering that patients with TNBC who were from other age groups had a higher mortality rate than those with other subtypes, patients younger than 40 years might have poor prognosis regardless of the subtype. This finding was also consistent with the results of the previous studies. A cohort study of patients with newly diagnosed BC from one of eight National Comprehensive Cancer Network centers in the United States found that progression of TNBC did not differ according to age group. However, patients with luminal BC who were younger than 40 years had higher risk of BC-specific mortality than patients in other age groups.^27^ Another study, using the Norwegian cancer registry, found that patients younger than 40 years and older patients (70 to 89 years) had higher BC-specific mortality than those 50 to 59 years old regardless of subtype.^28^ They also found that the influence of age was commonly observed in patients with luminal subtype.^28^ In our study, among patients younger than 40 years, those with TNBC had a 1.41-times higher risk of mortality than patients with ER+ or PR+ and HER2− BC, but this association was not statistically significant. In other age groups, the mortality rate among patients with TNBC was two-fold higher than among those with luminal type. Accordingly, we expected that patients with ER+ or PR+ and HER2− BC who were younger than 40 years had a relatively poor prognosis. Therefore, young patients with ER+ or PR+ and HER2− BC need more delicate therapeutic strategies to reduce disease recurrence and mortality. Furthermore, a recent genomic study showed that younger patients expressed ER at lower levels along with weaker expression signature for ER signaling, suggesting that their tumors were less dependent on estrogen signaling.^29^ The TAILORx (Trial Assigning Individualized Options for Treatment) clinical trial also suggested that younger premenopausal women with hormone receptor–positive BC, defined as ER+ or PR+ and HER2– BC, BC benefitted more from adjuvant chemotherapy and had higher disease-free survival rates than did women older than 50 years.^30^ Moreover, premenopausal women whose estrogen level did not significantly reduce after receiving endocrine treatment had higher BC-specific mortality.^31^ Therefore, delicate endocrine therapy and other systemic treatments for younger patients can improve survival.

In our study, patients who did not receive adjuvant treatment had worse progression. Patients who did not receive chemotherapy had approximately a two-times higher risk of mortality than patients who received chemotherapy, and patients who did not receive hormone therapy had a five-fold higher risk of mortality than patients who did receive this hormone therapy. Among the patients with HER2+ BC, those who did not receive targeted therapy had worse progression than those who did. These findings are consistent with those of previous studies. Many studies already demonstrated that adjuvant treatment is effective in improving survival.^32,33^ In our study, a substantially less proportion of patients older than 60 years than younger patients received chemo- and radiation therapy, and patients who did not receive hormone therapy had a 15-times higher risk of mortality than patients who did. Traditionally, the cutoff age of 65 years was considered to define elderly patients, who are considered vulnerable to standard adjuvant chemotherapy. According to a previous study, physicians were less likely to treat elderly women with adjuvant therapy after curative surgery,^34^ and older patients might be less likely to receive adjuvant therapy because they do not feel the necessity of additional treatment^35^ or because they have concerns about potential adverse effects.^36^ In fact, treatment-related symptoms are also a frequent reason for discontinuing therapy (20%).^37^ Older patients might be less likely to receive adjuvant therapy considering the potential adverse effects, resulting in poor progression. However, age should not be used as the sole criterion in deciding a therapeutic regimen for patients with BC.^4^ Instead, the estimated absolute benefit, life expectancy, tolerance, and performance of each patient should be considered. Meanwhile, all therapeutic regimens may need to be adapted for the elderly patients to minimize toxicity and achieve favorable long-term outcomes.^5^ Moreover, patients need to be informed about possible benefits of adjuvant therapy and hormonal therapies. Provision of education and supportive care would also be meaningful for elderly patients to comply with planned therapy.

The current study had some limitations. First, behavioral risk factors associated with outcomes such as smoking, drinking, and exercise were not evaluated. Second, although we had relatively larger sample size compared with those of previous studies, we still had limited power to evaluate the risk factors in each age group. Third, because the registry was from a single institution in Seoul, Korea, the characteristics of study patients would be different from those at other institutions or other countries. Hence, the results of our study might not be generalizable to other patients with cancer in other settings. Despite these limitations, this is the first comprehensive modern registry to include all subtypes and treatment information, presenting valuable information on the clinical characteristics and outcomes of patients with BC by age groups.

Altogether, we confirmed a strong J-shaped association between age and mortality, presenting worse clinical outcomes of young and old patients. We also confirmed the impact of stage at diagnosis, subtype, and adjuvant treatment on outcomes of BC regardless of age. This study suggested a possible benefit of personalized BC screening examination and precise and active treatment strategies to reduce BC mortality. Yet, considering that the BC epidemiology in Korea differs from that in Western countries,^2,38,39^ additional multicenter studies are warranted to develop a more precise treatment guideline by age group.
